# Phase II study of metformin for reduction of obesity-associated breast cancer risk: a randomized controlled trial protocol

**DOI:** 10.1186/s12885-016-2551-3

**Published:** 2016-07-19

**Authors:** Jessica A. Martinez, Pavani Chalasani, Cynthia A. Thomson, Denise Roe, Maria Altbach, Jean-Philippe Galons, Alison Stopeck, Patricia A. Thompson, Diana Evelyn Villa-Guillen, H-H. Sherry Chow

**Affiliations:** The University of Arizona Cancer Center, 1515 N Campbell Ave; Rm 2964B, Tucson, AZ 85724 USA; Department of Nutritional Sciences, The University of Arizona, Tucson, AZ USA; Department of Epidemiology and Biostatistics, The University of Arizona, Tucson, AZ USA; Department of Medical Imaging, University of Arizona, Tucson, AZ USA; Department of Medical Hematology/ Oncology, Stony Brook University, Stony Brook, NY USA; Department of Pathology, Stony Brook University, Stony Brook, NY USA

**Keywords:** Metformin, Breast cancer prevention, Breast density, Biomarkers, Metabolic syndrome, Metabolomics

## Abstract

**Background:**

Two-thirds of U.S. adult women are overweight or obese. High body mass index (BMI) and adult weight gain are risk factors for a number of chronic diseases, including postmenopausal breast cancer. The higher postmenopausal breast cancer risk in women with elevated BMI is likely to be attributable to related metabolic disturbances including altered circulating sex steroid hormones and adipokines, elevated pro-inflammatory cytokines, and insulin resistance. Metformin is a widely used antidiabetic drug that has demonstrated favorable effects on metabolic disturbances and as such may lead to lower breast cancer risk in obese women. Further, the anti-proliferative effects of metformin suggest it may decrease breast density, an accepted biomarker of breast cancer risk.

**Methods/design:**

This is a Phase II randomized, double-blind, placebo-controlled trial of metformin in overweight/obese premenopausal women who have elements of metabolic syndrome. Eligible participants will be randomized to receive metformin 850 mg BID (*n* = 75) or placebo (*n* = 75) for 12 months. The primary endpoint is change in breast density, based on magnetic resonance imaging (MRI) acquired fat-water features. Secondary outcomes include changes in serum insulin levels, serum insulin-like growth factor (IGF)-1 to insulin-like growth factor binding protein (IGFBP)-3 ratio, serum IGF-2 levels, serum testosterone levels, serum leptin to adiponectin ratio, body weight, and waist circumference. Exploratory outcomes include changes in metabolomic profiles in plasma and nipple aspirate fluid. Changes in tissue architecture as well as cellular and molecular targets in breast tissue collected in a subgroup of participants will also be explored.

**Discussion:**

The study will evaluate whether metformin can result in favorable changes in breast density, select proteins and hormones, products of body metabolism, and body weight and composition. The study should help determine the potential breast cancer preventive activity of metformin in a growing population at risk for multiple diseases.

**Trial registration:**

ClinicalTrials.gov Identifier: NCT02028221. Registered on January 2, 2014. Grant #: 1R01CA172444-01A1 awarded on Sept 11, 2013.

## Background

### Obesity and breast cancer

It is predicted that by 2030 there will be 65 million more obese adults in the USA which will contribute to 492,000–669,000 additional cases of cancer [1]. In addition to cancer, high body mass index (BMI) is a major risk factor for a number of chronic diseases, including type 2 diabetes and cardiovascular diseases [2–5]. For postmenopausal breast cancer, a high BMI has been reported to increase risk by 30–50 % [6]. The increased risk for postmenopausal breast cancer in women with high adiposity is likely attributed to multiple metabolic disturbances that occur with overweight and obesity including, but not limited to, altered circulating sex steroid hormones, hyperinsulinemic insulin resistance, altered expression and secretion of adipokines from adipose tissue, increased production of pro-inflammatory cytokines, and increased oxidative stress. While intensive weight loss programs can be moderately successful [7], two-thirds of the U.S. adult women are still overweight or obese [8]. Thus, identification of chemoprevention agents that correct the metabolic dysregulation associated with overweight and obesity, even in the absence of weight loss, is a highly attractive strategy for lowering breast cancer risk.

### Metformin for reduction of obesity-associated breast cancer risk

Metformin is a biguanide indicated as an adjunct to diet and exercise to improve glycemic control in adults and children with type 2 diabetes mellitus. It has been used worldwide for over 40 years to treat type 2 diabetes, and was approved in the United States by the Food and Drug Administration as an antidiabetic in 1995 [9]. It is also commonly used off-label for metabolic syndrome [10] as well as polycystic ovary syndrome (PCOS) [11], a condition also characterized by metabolic disturbance. The major mechanism of metformin action in vivo involves suppression of hepatic gluconeogenesis and glucose output, which is associated with a decline in circulating glucose concentration and a secondary decline in insulin levels [12].

Metformin exerts favorable effects on multiple metabolic disturbances that may lead to reduction of breast cancer risk. In diabetics, metformin treatment resulted in favorable changes in circulating levels of leptin and adiponectin [13, 14]. In women with PCOS, metformin has been shown to reduce circulating insulin levels (reviewed by [11]), increase insulin-like growth factor binding protein (IGF-BP) 1 [15, 16], decrease serum testosterone and androstenedione levels [17, 18], and increase serum sex steroid hormone binding globulin [17, 18]. Reduction in serum insulin levels was also observed in non-diabetic breast cancer patients with metformin treatment [19]. Metformin has also been used to treat weight gain induced by antipsychotic medications [20]. It has also been investigated for weight loss in overweight but otherwise healthy individuals with success in some but not all studies, however, these trials had small sample size and design limitations that likely contributed to the inconsistent results across studies (reviewed by [21]).

In addition to indirect effects, metformin may exert a direct effect in mammary tissue through the activation of the AMP-activated protein kinase (AMPK) signaling pathway, leading to an antiproliferative effect and induction of apoptosis [22–25]. However, metformin is an organic cation at physiological pH. Its cellular uptake generally requires the presence of the cell surface transporters such as organic cation transporters (OCT) 1, 2 or 3. It is not known whether normal human mammary tissue expresses the transporters for metformin to exert its direct effect. In a recent neoadjuvant trial, however, expression of the OCT1 transporter was detected in breast tissue [26].

### Epidemiological and clinical evidence of metformin for breast cancer prevention

Some, but not all case control and cohort studies investigating the relationship between diabetes and cancer have found that treatment with metformin appears to substantially reduce the risk for development of cancer in individuals diagnosed with diabetes [27, 28], including lower risk for breast cancer [29–32]. Some evidence also suggests a role for metformin in prolonging breast cancer survival [33, 34]. Given the retrospective nature of these studies and the possibility that the comparison treatments (such as sulfonylureas or exogenous insulin) may increase risk, randomized, placebo-controlled intervention trials are needed to assess the breast cancer preventive activity of metformin. It is important to note that accumulating evidence suggests that type 2 diabetes and obesity share biological mechanisms for their association with postmenopausal breast cancer which include pathways targeted by metformin (reviewed by [35]). Therefore, metformin could modify breast cancer risk in people with elevated BMI independent of a diagnosis of diabetes.

Recent window-of-opportunity neoadjuvant trials reported clinical activity of metformin in non-diabetic women with operable invasive breast cancer. Two small non-placebo controlled trials showed decreased tumor cell proliferation following a short-term metformin intervention prior to surgery [36, 37]. A considerably larger randomized, placebo-controlled trial (*N* = 200) did not show an overall decrease in tumor cell proliferation after 4 weeks of metformin intervention [38]. However, subgroup analyses showed a decrease in tumor cell proliferation and serum insulin levels in women with high waist/hip ratio or insulin resistance, suggesting the importance of the metabolic characteristics of the study population [38]. In a recent small trial with early stage breast cancer patients, metformin administration significantly decreased expression of the insulin receptor, phophorylated protein kinase B (PKB), and phosphorylated extracellular signal-regulated kinase 1/2 (ERK 1/2) in tumor tissue [26], suggesting a therapeutic benefit.

An ongoing, large Phase III randomized trial (NCIC CTG MA.32) in over 3,500 early stage breast cancer patients aims to determine whether adding metformin to standard adjuvant therapy will improve invasive disease free survival. Recently published interim data from this trial shows a statistically significant decrease in weight, insulin, glucose, leptin, and CRP at six months in the metformin arm verses placebo [39]. The outcomes of this trial will be important in assessing metformin’s effectiveness on recurrence and survival in breast cancer patients. However, the majority of the study population has received standard adjuvant radiation, endocrine treatment, trastuzumab or other biologics or bisphosphonates prior to or during study treatment, or adjuvant or neoadjuvant chemotherapy prior to study treatment. In general, applicability of the data generated from this trial to a potential role for metformin as a single agent for breast cancer risk reduction in at-risk women with no prior breast cancer history is unclear and requires further research. Our study is one of the first placebo controlled trials to evaluate the effect of metformin on breast cancer risk factors in at risk healthy women.

### Overall study goals

The overall objective of our study is to evaluate the effect of metformin on biomarkers that have shown strong clinical and scientific relevance to breast cancer risk and have high potential to be modulated by metformin in a cohort of overweight premenopausal women. The primary study aim is to determine the effect of metformin on breast density. Studies suggest that women with dense tissue in more than 60–75 % of the breast are at 4-6-fold greater risk of developing breast cancer than those with no dense tissue [40–42]. We hypothesize that metformin intervention will reduce breast density because metformin has been shown to decrease breast cell proliferation and modulate the IGF axis [15, 16, 36, 37], which have both been associated with variation in breast density [43–46]. Breast density will be assessed by magnetic resonance imaging (MRI)-acquired fat-water features, a novel, three-dimensional measure of breast density that will provide more sensitive and quantitative detection of changes in breast density than mammographic measure. The study is enrolling premenopausal women instead of postmenopausal women because premenopausal women have a higher breast density at baseline, which may allow for more notable detection of breast density reduction by metformin. Further, this study population is at increased risk for future postmenopausal breast cancer.

This study is one of the first placebo-controlled, randomized trials to determine metformin effects on multiple metabolic disturbances as well as anthropometric measures in non-diabetic women. In addition, we will explore the application of metabolomics analysis to both plasma and nipple aspirate fluid as a systems biology approach to assess the potential chemopreventive mechanisms of metformin and to understand metabolic features affecting systemic and tissue markers of breast cancer risk. Metabolomic profiling in fluid expressed from the breast may provide information about the breast microenvironment that may not be reflected in plasma. Further, we will also have the opportunity to explore breast-tissue level effects of metformin in a subset of women. Thus, we will have the unique opportunity to explore the associations between metabolic features in nipple aspirate fluid and plasma with markers of breast cancer risk in both plasma and in breast tissue to further our understanding of the underlying biochemical mechanisms affecting these risk markers. Findings from this study will add insight into whether metformin can be used clinically as a pharmacological approach for breast cancer risk reduction in premenopausal women with high adiposity.

## Methods/design

### Objectives

#### Primary objective

The primary objective is to determine the effect of metformin intervention on breast density assessed by MRI acquired fat-water features.

#### Secondary objectives

The secondary objectives are to determine the effect of metformin intervention on metabolic disturbances and body weight/composition, and to apply metabolomics as a systems biology approach to assess the chemopreventive mechanisms of metformin.

### Exploratory objectives

An exploratory objective is to determine the effect of metformin intervention on tissue architecture as well as cellular and molecular targets in breast tissue collected in a subgroup of participants. We will also explore whether metformin-induced metabolic changes correlate with changes in markers of breast cancer risk or side effects.

### Study design

**Fig. 1:**
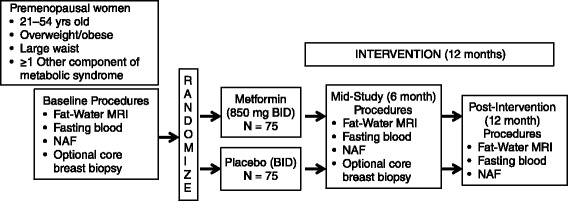
Overall study design. MRI: magnetic resonance imaging; NAF: nipple aspirate fluid

This is a Phase II double-blind, randomized, placebo-controlled trial of metformin to determine its potential effects on recognized and putative markers of breast cancer risk in overweight or obese premenopausal women with elements of metabolic syndrome. Figure 1 illustrates the overall study design.

### Participant recruitment

This is a single institution trial conducted at the University of Arizona Cancer Center (UACC), USA. Women will be recruited through clinic offices, television and radio advertising, printed flyers, social media, and word of mouth.

### Eligibility criteria

#### Inclusion criteria

- Premenopausal women
- 21–54 years of age
- No change in menstrual patterns for the past 6 months preceding the time of registration
- Have a body mass index of 25 kg/m^2^ or greater
- Large waist circumference:
  - ○ ≥88 cm (≥35 in.) or
  - ○ ≥80 cm (≥31 in.) for Asian Americans, individuals with PCOS, or individuals with non-alcoholic fatty liver disease
- Have at least one other component of metabolic syndrome [47]:
  - ○ Elevated triglycerides (≥150 mg/dL (1.7 mmol/L)) or on drug treatment for elevated triglycerides,
  - ○ Reduced high-density lipoprotein cholesterol (<50 mg/dL (1.3 mmol/L)) or on drug treatment for reduced high-density lipoprotein cholesterol,
  - ○ Elevated blood pressure (≥130 mmHg systolic blood pressure or ≥85 mmHg diastolic blood pressure) or on antihypertensive drug treatment in a patient with a history of hypertension, or
  - ○ Elevated fasting glucose (≥100 mg/dL)
- Mammogram negative for breast cancer within the 12 months preceding the time of registration for women ≥ 50 years of age
- Ability to understand and willingness to sign a written informed consent document

#### Exclusion criteria

- Postmenopausal women
  - ○ Amenorrhea for at least 12 months (preceding the time of registration), or
  - ○ History of hysterectomy and bilateral salpingo-oophorectomy, or
  - ○ At least 55 years of age with prior hysterectomy with or without oophorectomy, or
  - ○ Age 35 to 54 with a prior hysterectomy without oophorectomy OR with a status of ovaries unknown with documented follicle-stimulating hormone level demonstrating elevation in postmenopausal range
- Women who are pregnant, planning pregnancy within the next year, or lactating/breastfeeding
- On treatment with any drug for diabetes
- Have uncontrolled intercurrent illness including, but not limited to, ongoing or active infection, symptomatic congestive heart failure, unstable angina pectoris, cardiac arrhythmia, or any illness that would limit compliance with study requirements
- Have received chemotherapy and/or radiation for any malignancy (excluding non-melanoma skin cancer and cancers confined to organs with removal as only treatment) in the past 5 years (preceding the time of registration)
- Have received other investigational agents within the past 3 months (preceding the time of registration)
- Have a history of lactic acidosis or risk factors for lactic acidosis
- Have renal disease or dysfunction (creatinine ≥ 1.4 mg/dL)
- Have hepatic dysfunction (bilirubin > 1.5 × ULN unless with Gilberts syndrome or AST/ALT > 3 × ULN)
- Have a history of alcoholism or high alcohol consumption (average of > 3 standard drinks/day)
- Have a history of allergic reactions to metformin or similar drugs
- Have a history of severe claustrophobia
- Have electrically, magnetically, or mechanically activated implants including cardiac pacemaker, cochlear implants, magnetic surgical clips or prostheses
- Have breast implants

### Randomization

Randomization is performed using computer-generated random permuted blocks. To retain the blind, metformin and placebo supplies are identified by the randomization number. The study staff will dispense the product to subjects based on the assigned randomization number. None of the staff interacting with subjects will know the link between randomization number and actual product. The code that identifies the product will be kept by the study statistician or the designated data manager.

Unblinding is not expected to occur until all subjects complete the intervention and data entry is complete. If deemed medically necessary, study agents may be unblinded by the Principle Investigator in consultation with the Medical Director in the event of a serious adverse event (SAE).

### Agent administration

Subjects will be on agent intervention for 12 months (52 ± 4 weeks). For the first four weeks, 1 metformin (850 mg) or placebo tablet will be taken once daily with food. For the remaining treatment period, metformin or placebo tablets will be taken twice daily with food.

### Concomitant medications

Participants may not use non-study metformin or other biguanides while on study. Medications with potential interaction with metformin (e.g. cationic drugs) will be carefully reviewed and monitored closely while subjects are on study. All medications (prescription and over-the-counter), vitamin and mineral supplements, and/or herbs taken by the participant will be documented on the concomitant medication case report form and include: start and stop date, dose and route of administration, and indication.

### Schedule of study procedures

A schedule of study procedures is presented in Table 1.

**Table 1:** Schedule of Study Events

Study Events	Consenting/Screening	Baseline/Randomization	Intervention (12 month (52 ± 4 weeks))								
			Months 1–3	Month 3 Visit (13 ± 2 weeks)	Months 4–6	Month 6 Visit (26 ± 4 weeks)	Months 7–9	Month 9 Visit (39 ± 2 weeks)	Months 10 –12	Month 12 Visit (52 ± 4 weeks)	Follow up
Consent, med records release form	x										
Medical history, performance status	x										
Anthropometric measurements	x	x				x				x	
Vital signs (Temp, BP, Pulse)	x	x		x		x		x		x	
Concomitant meds	x	x		x		x		x		x	
Breast cancer risk questionnaire	x										
Menstrual pattern/cycle review	x	x		x		x		x		x	
Menstrual cycle diary	x	x	x	x	x	x	x	x	x	x	
Fasting blood (CBC/Diff, CMP, lipids, FSH^a^, estradiol^a^)	x									x	
Fasting blood for research endpoints		x				x				x	
Urine pregnancy test	x	x		x		x		x		x	
Urine for research endpoints		x				x				x	
AFFQ, AAFQ^b^		x								x	
Nipple aspirate fluid (NAF) collection		x				x				x	
Breast MRI^c^		x				x				x	
Core biopsy, if consented		x				x					
Final eligibility assessment		x									
Randomization		x									
Dispense study agent		x				x					
Intake calendar			x	x	x	x	x	x	x	x	
Adverse events diary		x	x	x	x	x	x	x	x	x	x
Adverse events review		x		x		x		x		x	x
Return study agent						x				x	
Compliance assessment				x		x		x		x	
Case report form completion	x	x		x		x		x		x	x
Telephone/email contact^d^			x		x		x		x		

^a^FSH (follicle-stimulating hormone) and/or estradiol at screening for women with uncertain menopausal status

^b^AFFQ: Arizona Food Frequency Questionnaire; AAFQ: Arizona Activity Frequency Questionnaire

^c^Women who cannot fit into the MRI scanner due to the large body size will continue the study and undergo all other study procedures

^d^Study personnel will contact subjects within a week after study agent has been initiated and within a week following scheduled dose increase to assess compliance and any potential problems. Additional periodic telephone or email contact will occur between study visits and as needed to review study procedures, adverse events, concomitant medications, and to address any subject concerns

#### Consent to participate

Potential subjects will present to clinic for a detailed discussion of the protocol with the study coordinator. Signed informed consent will be obtained prior to any study-related activities or procedures being conducted. Subjects are registered onto the protocol on the day of consent. Participants will then undergo the following procedures for screening.

- Review of medical history and medication usage history
- Collection of demographic information (age, race/ethnicity)
- Collection of anthropometric measurements (weight, height, waist circumference, waist-hip ratio)
- Collection of breast cancer risk information (family and personal history of breast cancer, age at menarche, parity, and prior breast biopsy)
- Collection of information on menstrual patterns/cycles
- Measures of vital signs (temperature, blood pressure and pulse).
- A fasting blood sample for complete blood count with differential (CBC/w diff), comprehensive metabolic panel (CMP), lipids, and follicle-stimulating hormone and/or estradiol for women with uncertain menopausal status
- Urine pregnancy test

#### Baseline procedures

Participants who meet all selection criteria will undergo the following baseline procedures. When feasible, these procedures will be scheduled in the midluteal phase of the menstrual cycle.

- Anthropometric measurements
- A fasting blood for research biomarkers
- Collection of urine for urine pregnancy test and research tests
- Vital signs
- Update information on menstrual patterns/cycles
- Update medication usage
- Completion of the Arizona Food Frequency Questionnaire (AFFQ) to measure usual dietary intake and the Arizona Activity Frequency Questionnaire (AAFQ) to assess usual physical activity
- Collection of nipple aspirate fluid (NAF)
- MRI assessment of breast density – MRI is performed on a Seimens 3T MRI system using a 16 channel breast MRI coil system. Fat-water maps will be obtained using a multi-point Gradient Echo DIXON imaging method developed by Siemens [48–50]. Women who cannot fit into the MRI scanner due to large body size will continue the study and undergo all other study procedures
- Optional core needle biopsy - for participants who consent to this optional procedure, the medical specialist will use a 14-gauge needle under ultrasound guidance to obtain up to 8 tissue cores from areas of high density in one of the breasts

Following completion of baseline evaluation, participants will be randomized to receive metformin or placebo for 12 months. Participants will be asked to keep an adverse event (AE) diary and menstrual calendar throughout the study. In addition, participants will be provided with an intake calendar for recording medication usage. Study personnel will contact study participants within a week after study agent has been initiated and within a week following the scheduled dose increase to assess compliance and any potential problems.

#### 6-month visit procedures

Participants will return at month 6 (26 ± 4 weeks) to undergo the following procedures:

- Collection of urine for urine pregnancy test and research tests
- Vital signs
- Update information on menstrual patterns/cycles
- Update medication usage
- Side effect evaluation
- Return unused pills
- Receive a new supply of study medication
- Anthropometric measurements
- Collection of a fasting blood and NAF sample for research biomarkers
- MRI assessment of the breast
- Optional breast core biopsy

When feasible, the month 6 procedures will be scheduled in the mid-luteal phase of the menstrual cycle.

#### Completion of study intervention procedures

Participants will be instructed to take the study agent until the day of the last study procedure. At the end of the 12-month agent intervention (52 ± 4 weeks), participants will return to the clinic to undergo the following procedures:

- Collection of urine for urine pregnancy test and research tests
- Vital signs
- Update information on menstrual patterns/cycles
- Update medication usage
- Return unused pills
- Side effects evaluation
- Anthropometric measurements
- A fasting blood for CBC/CMP/lipids and research biomarkers
- NAF collection
- MRI assessment of breast density
- Completion of AFFQ and AAFQ

When feasible, the month 12 procedures will be scheduled in the midluteal phase of the menstrual cycle.

After completing the study intervention, participants will be followed for 2 weeks for any adverse reactions.

#### Additional visit procedures

Throughout the study, study personnel will contact study participants as needed between clinic visits to assess compliance and any potential problems. In addition to clinic visits with study endpoint collections, participants will return to the clinic at month 3 (13 ± 2 weeks) and month 9 (39 ± 2 weeks) after initiation of agent intervention in order to conduct a urine pregnancy test, obtain vital signs, update menstrual cycle information, update medication usage, and to evaluate side effects to ensure safety as well as participant compliance.

### Safety

All AEs will be assessed according Common Terminology Criteria for Adverse Events Version 4, followed according to good medical practices, and documented.

### Data and safety monitoring

The University of Arizona Cancer Center (UACC) Data and Safety Monitoring Board (DSMB) will provide ongoing oversight for this trial. Routine monitoring will be provided by the UACC Quality Assurance/Quality Control (QA/QC) Program to ensure that the investigation is conducted according to protocol design and regulatory requirements. The Principal Investigator will ensure the accuracy, completeness, legibility and timeliness of the data reported in the Case Report Form (CRF). Source documentation supporting the CRF data will indicate the participant’s participation in the trial and should document the dates and details of study procedures, AEs, and participant status.

### Details of power calculation and sample size

Our sample size ensures adequate power to test all primary and secondary outcomes. We assume an initial sample size of 75 women per group and allow the possibility of up to 20 % dropout for the 12-month follow-up measurement, resulting in at least 60 evaluable women per group. To determine the detectable effect size, we use a simplified comparison of change between 12 months versus baseline, assuming a two-sided α of 0.05 and 80 % statistical power. For the fat-water MRI-derived change in breast density, we will be able to detect a decrease of 0.516 standard deviation (SD) units in the metformin treated women, equivalent to a 10 % decrease in breast density as determined by standard mammography. For the secondary outcomes (such as systemic hormones, cytokines, and weight), we will also be able to detect a difference of 0.516 SD units. For metabolomic analysis that compares thousands of metabolites, we approximated the effect size by setting a more stringent alpha level of 0.005. For plasma metabolomic analysis we will be able to detect an effect size of 0.667 SD units, while for NAF metabolomic analysis we will be able to detect an effect size of 0.848 SD units (assuming at least 65 % of the women will provide sufficient NAF).

### Statistical analysis

The primary study endpoint is to compare the change in breast density as measured by fat-water MRI (when measured at baseline, 6 and 12 months) between metformin and placebo groups. Additional endpoints such as systemic hormone and cytokines, body weight, waist circumference, and waist-hip ratio will be measured at baseline, 6 and 12 months. Analysis for all study endpoints will be based on a linear mixed-effects model for the observed values across time, to adjust for the correlation among measurements within the same woman. The main effects in the model will be time (0, 6, 12), treatment group (metformin versus placebo), and the interaction between time and treatment group. The time parameter tests if there is a change among placebo treated women, while the group-by-time interaction tests if the change in metformin treated women differs from that in the placebo group. We expect that a simpler covariance structure, such as compound symmetry, will be adequate for repeated measurements within the same woman (since the measurements are equally spaced). Alternatively, we will compare correlation structures for the longitudinal measurements using Akaike’s information criterion. All endpoints will be assessed for normality, and transformations (such as a logarithmic transformation) will be used as necessary to reduce skewness prior to statistical analysis.

For the metabolomic aim, pre- to post-intervention changes in all detectable compounds will be determined and compared between treatment groups using two-sample t-tests. An estimate of the false discovery rate (*q*-value) will be calculated [51] to adjust for the multiple comparisons that normally occur in metabolomic-based studies. A low *q*-value (*q* ≤ 0.10) is an indication of high confidence in a result. While a higher *q*-value indicates diminished confidence, it does not necessarily rule out the significance of a result. Other lines of evidence may be taken into consideration when determining whether a result merits further scrutiny. Such evidence may include a) significance in another dimension of the study, b) inclusion in a common pathway with a highly significant compound, or c) residing in a similar functional biochemical family with other significant compounds.

No interim statistical analyses are planned for this Phase II trial. Accrual, data collection, and any AEs will be monitored on a regular basis. The final dataset will be available to all study investigators.

## Discussion

The trial was activated in March of 2014. As of May, 2016, we have consented 152 women. Fifty were ineligible, 5 dropped out prior to agent intervention, 97 initiated agent intervention. It is expected that we will complete accrual by the summer of 2017 and participant treatment by the summer of 2018. Over 80 % of the accrued participants have agreed to undergo the optional breast biopsy. Findings from this study will be published in a peer-reviewed scientific journal and will have wide public health impact because of the growing overweight and obese populations at risk for multiple diseases. With its demonstrated effect in reducing the incidence of diabetes in high-risk adults [52], metformin would have a high level of acceptance and uptake in at risk women with high adiposity if it has also been shown to exert favorable activities in breast cancer risk reduction. Considering the challenges in maintaining a healthy life style by the majority of the general public and the pleiotropic activities of metformin for multiple metabolic disorders, metformin could be developed as an integrated pharmacological approach for at risk women with high adiposity for prevention of multiple chronic diseases including breast cancer.

## Abbreviations

AAFQ, Arizona Activity Frequency Questionnaire; AE, adverse event; AFFQ, Arizona Food Frequency Questionnaire; AMPK, AMP-activated protein kinase; CBC/w diff, complete blood count with differential; CMP, comprehensive metabolic panel; DSMB, Data and Safety Monitoring Board; ELISA, enzyme-linked immunosorbent assay; HPLC, high performance liquid chromatography; IGF-BP, insulin-like growth factor binding protein; MRI, magnetic resonance imaging; MS, mass spectrometry; NAF, nipple aspirate fluid; NCI, National Cancer Institute; PCOS, polycystic ovary syndrome; QC, quality control; SAE, serious adverse event; SD, standard deviation; UACC, University of Arizona Cancer Center
